# Controlling the characteristics of injected and accelerated electron bunch in corrugated plasma channel by temporally asymmetric laser pulses

**DOI:** 10.1038/s41598-022-11955-6

**Published:** 2022-05-17

**Authors:** M. Sedaghat, A. Amouye Foumani, A. R. Niknam

**Affiliations:** Laser and Plasma Research Institute, Shahid Beheshti University, 1983969411 Tehran, Iran

**Keywords:** Plasma-based accelerators, Laser-produced plasmas

## Abstract

In laser-driven plasma wakefield accelerators, the accelerating electric field is orders of magnitude stronger than in conventional radio-frequency particle accelerators, but the dephasing between the ultrarelativistic electron bunch and the wakefield traveling at the group velocity of the laser pulse puts a limit on the energy gain. Quasi-phase-matching, enabled by corrugated plasma channels, is a technique for overcoming the dephasing limitation. The attainable energy and the final properties of accelerated electron beams are of utmost importance in laser wakefield acceleration (LWFA). In this work, using two-dimensional particle-in-cell simulations, the effect of the driving pulse duration on the performance of quasi-phase-matched laser wakefield acceleration (QPM-LWFA) is investigated. It is observed that for a pulse duration around half the plasma period, the maximum energy gain of the beam electrons finds its peak value. However, the results show that for a pulse of that duration the collimation of the bunch is much worse, compared to the case where the pulse duration is twice as long. Furthermore, the dynamics of the laser pulse and the evolution of the quality of the externally-injected electron bunch are studied for a symmetric pulse with sine-squared temporal profile, a positive skew pulse (i.e., one with sharp rise and slow fall), and a negative skew pulse (i.e., one with a slow rise and sharp fall). The results indicate that for a laser pulse with an appropriate pulse length compared with the plasma wavelength, the wakefield amplitude can be greatly enhanced by using a positive skew pulse, which leads to higher energy gain. Initially, this results from the stronger ponderomotive force associated with a fast rise time. Later, due to the distinct evolution of the three pulses with different initial profiles, the wakefield excited by the positive skew pulse becomes even stronger. In our simulations, the maximum energy gain for the asymmetric laser pulse with a fast rise time is almost two times larger than for the temporally symmetric laser pulse. Nevertheless, stronger focusing and defocusing fields are generated as well if a positive skew pulse is applied, which degrade the collimation of the bunch. These results should be taken into account in the design of miniature particle accelerators based on QPM-LWFA.

## Introduction

Laser wakefield acceleration (LWFA)^1,2^ is a scheme which utilizes strong laser-induced plasma waves to accelerate electrons to relativistic energies over a very short distance^3,4^. The ultimate goal of research on this scheme is production of inexpensive, compact and lightweight accelerators which are comparable with bulky traditional accelerators in terms of energy gain and beam quality^5,5–8^. LWFA has been demonstrated and explored in numerous experiments so far and is a very active research area at present^9–11^. These experiments have mainly been carried out in the so-called “bubble regime”^9,11–13^. Quasi-mono-energetic (low energy-spread), collimated GeV electron bunches with charges in the pC range have been produced in centimeter-scale acceleration length by using laser pulses with peak powers ranging from tens of TW to PW^10,14,15^. These beams are usable as compact radiation sources in the X-ray and $\gamma$-ray region^16–22^. Although petawatt lasers produce high intensity pulses, the rate of pump depletion and etching is greater at high intensities^23,24^. Moreover, in high power laser systems, due to technical obstacles, the frequency of optical pumping is low, thus the repetition rate of such lasers is as low as $\sim$1–10 Hz. This reduces the attractiveness of conventional LWFA for applications like advanced X-ray radiography, which demand high average output^20,25–27^. Therefore, there exists much interest in accelerating relativistic electrons within the linear regime of LWFA, in which lower peak power lasers ($<1$ TW) with higher repetition rates (up to kHz) and pulse energies of several mJ are used^25,26^.

In general, there are three disruptive processes the control of which is of particular importance in LWFA, namely dephasing, pump depletion, and diffraction^8,11,15,23^. The phase velocity of a laser-driven wakefield is approximately equal to the group velocity of the laser pulse in the plasma, which is somewhat lower than the speed of light. Thus, the highly relativistic electrons outpace the plasma wave and eventually slip from an accelerating region into a decelerating region of the wakefield^2^. This process, known as dephasing, limits the acceleration length to the so-called dephasing length, which scales as $L_{\text {d}} \propto n_{0}^{-3/2}$, where $n_{0}$ is the plasma density^23^. Dephasing is an important limitation that must be overcome in order to achieve higher electron energy gains, particularly for accelerators operating with lower power lasers. Another limitation is the depletion of the energy of the laser pulse as it drives the wakefield. The laser pump depletion length, $L_{\text {dep}}$, is the length scale over which this depletion occurs, and it scales with the plasma density in the same manner as the dephasing length does^2,23^. Since the maximum accelerating field scales as $E_{\text {max}} \propto n_{0}^{1/2}$, the energy gain in LWFA, $\Delta \gamma \propto E_{\text {max}} L_{\text {d}} \propto n_{0}^{-1}$, can be increased by using a plasma of lower density. Decreasing the plasma density also increases the dephasing length and pump depletion length.

However, for optimum acceleration of electrons over long distances, which can be larger than tens of Rayleigh lengths, one has to ensure that the laser pulse is propagated up to the dephasing length with minimum diffraction. One solution is based on utilizing self-guiding, in which the combined effect of relativistic self-focusing and ponderomotive self-channeling guides the pulse^2,28^. This occurs in situations where the laser power is greater than the critical power, $P_{\text {crit}}=17{\omega _{0}}^2/{\omega _{\text {p}}}^2$ GW, in which $\omega _{\text {p}}$ and $\omega _{0}$ are the plasma frequency and laser frequency, respectively. For sub-TW laser pulses, in order to have self-guided propagation, it is essential to perform LWFA at high plasma densities^23,29,30^. For example, Goers et al. accomplished sub-TW LWFA by focusing 0.21 TW laser pulses onto hydrogen gas targets ($n_{\text {e0}} \ge 2 \times 10^{20}$ cm$^{-3}$)^26^. Another way to reduce diffraction is by using a larger spot size, as Rayleigh length increases with spot size, but this approach requires high pulse power. Diffraction can also be reduced by using a parabolic transverse density profile that has a minimum on the laser propagation axis. Such a structure can be matched to guide a gaussian laser mode without distortion in the low intensity limit over many Rayleigh lengths^2,31–33^.

Extending the energy gain beyond what the dephasing limitation dictates is a prime objective in the development of LWFA accelerators. One approach to overcoming the dephasing limitation is to link multiple LWFA stages in series^34–36^. The use of a new laser pulse at the beginning of each stage makes laser diffraction and depletion more manageable. However, in practice this approach faces severe practical challenges with regard to beam extraction and transfer to the next plasma stage, to which the new pulse must be coupled with femtosecond accuracy. In addition, this approach requires a high total laser energy. An alternative, more efficient approach is to manipulate the wakefield using density gradients within a single stage so that the electron stays in the accelerating region of the wakefield. In the present work, we adopt this approach and use a plasma channel having an axially-periodic density profile. Axially corrugated plasma channels have been successfully generated in experiments done by Layer et al.^37,38^. The laser-induced plasma wakefields in such a medium can be decomposed into spatial harmonics whose related phase velocities depend on the modulation period^39,40^. This property can be exploited in quasi-phase-matching-based LWFA (LWFA-QPM)^41,42^, in which by equating the density modulation period of the corrugated channel to the dephasing length, the phase velocity of an individual spatial harmonic is matched to the velocity of the accelerating electron beam, which is close to the speed of light, *c*. Consequently, the chosen individual spatial harmonic exerts an almost constant axial force on the relativistic electrons, while the time average of the axial forces from all other spatial harmonics is zero. Comparison of the longitudinal wakefields acting on an electron moving at nearly *c* in modulated and unmodulated channels shows that in a modulated channel because of the break in the symmetry of energy gain and loss between the acceleration and deceleration phases experienced by relativistic electrons, the wakefield performs net work on an electron even after it has traversed one dephasing length, while the integral of the axial field over a plasma period is zero in a uniform channel^41^. As a result, for acceleration using linear wakefields, this technique leads to energy gains several times greater than that from an equivalent uniform plasma channel. Moreover, using a preformed plasma channel allows the pulse to be guided without diffraction over distances in the order of centimeters, which is necessary to reach the desired energy gains.

The interaction of high-intensity laser pulses with underdense plasmas in LWFA is a complex process occurring in the relativistic regime. As the laser pulse propagates in the plasma medium, its shape evolves due to several nonlinear effects such as group velocity dispersion, self-phase modulation, and self-steepening^24,43–45^, as a result of which the temporal profile of the pulse becomes asymmetric. This asymmetry affects the evolution of the pulse as well as the maximum gain and quality of the injected electron beam. Similarly, LWFA is drastically affected by the initial properties of the laser pulse. In this paper, we propose a simple method to enhance the performance of QPM-LWFA by controlling the waveform of the driving laser pulse.

It has been observed that the temporal profile of the driving laser pulse plays an important role in wakefield generation^46–50^. Previous experimental investigations of the impact of asymmetries in pulse envelope and also frequency chirps on plasma wake excitation performed within the self-modulated LWFA regime have shown significant enhancement of the electron energy and total charge for sharp rising asymmetric pulses with positive chirps^51,52^.

In addition, experimental and theoretical studies in the bubble regime show that frequency chirped and shaped laser pulses can be used to adjust the self-injection rate, beam charge and the output energy of LWFA in the bubble regime^47,48,50,53^. It is found that a positively chirped laser pulse, in which the frequency is lower at the front of the pulse, can create stronger and more stable plasma waves than a negatively chirped or a chirp-free laser pulse. The reason is that when the pulse is positively chirped, the ponderomotive force is stronger at its front. By generating stronger plasma waves, which leads to a decrease in the effect of laser fluctuations, a positively chirped laser pulse also improves the stability of the electron beam^48^. Similarly, temporal laser pulse shapes with a sharp rising front (positive skew) are able to drive larger wakefields, leading to higher electron beam charge and energy^47,54–56^. Such asymmetric laser pulses have been generated experimentally in chirped-pulse amplification laser systems by detuning the laser pulse compressor. Electron beams with stable and small pointing angles have been reported to be obtained using asymmetric laser pulses^57^. In ionization-induced injection, a negatively skewed laser pulse profile results in a lower energy spread compared to a positively skewed pulse, thus yielding higher bunch quality^58^.

In this study, through two-dimensional (2D) particle-in-cell (PIC) simulations with the code OSIRIS, the dependence of the final features of the accelerated bunch and its dynamics on the pulse duration and the asymmetries in the pulse envelope is investigated. It is observed that in QPM-LWFA, by selecting proper pulse length, higher energy gain can be achieved. Although further reduction of the laser pulse duration leads to generation of larger ponderomotive force, the use of a relatively short pulse may cause problems due to a short depletion length. Indeed, there must be a trade-off between the weak ponderomotive force available from a long pulse and the pump depletion effect which can cause rapid energy loss of a short pulse in a plasma. Furthermore, the simulations show that the skewness of the laser pulse influences both the quality and the maximum output energy of the electrons injected into a corrugated plasma channel. It is found that for otherwise identical initial parameters, a temporal pulse profile with fast rise time ($\le$ plasma period) can excite a wakefield of significantly larger amplitude in comparison to a profile with slow rise time or a symmetric profile, resulting in enhanced peak energy of the bunch electrons. Initially, though all the three temporal profiles have the same peak intensity, the pulse with fast rise time exerts greater ponderomotive force, which is responsible for the generation of a stronger wakefield. The simulations show that the three pulses evolve differently, the pulse with positive skew reaching higher maximum intensity compared to the other two pulses. The laser envelope asymmetries have also significant impact on the final properties of the accelerated bunch. Using positively skewed pulses leads to considerable emittance growth because of the stronger transverse wakefield they generate.

## Simulation model and parameters

To study the effect of laser pulse shape on the final properties of an externally injected electron beam of moderate initial energy within the framework of QPM-LWFA, we perform fully self-consistent 2D PIC simulations using the code OSIRIS^59^ which is a massively parallel electromagnetic PIC code. The simulation window which consists of 16384 $\times$ 512 grid points and has dimensions of 438 μm $\times$ 77 μm, moves at the speed of light in the same direction that the electron beam and the laser pulse travel. The number of superparticles per grid cell is 4 and 9 for the plasma and the electron beam, respectively. At the upper and lower longitudinal boundaries open-space boundary conditions are applied for both the electromagnetic fields and the particles.

In our simulations, the driving laser pulse has a central wavelength of $\lambda _{\text {L}}=800$ nm and is linearly polarized. It has a Gaussian transverse profile and a sine-squared temporal profile. The duration varies between $\sigma _{\text {FWHM}}=20$ fs and 40 fs in the simulations. The pulse is focused into a waist radius of $w_0=15$ μm. The magnitude of the normalized vector potential $a_0\equiv {eA}/{m_{\text {e}}c^2}$ is chosen to be 0.25, which corresponds to a low peak power of 0.5 TW. Here, *A*, *e* and $m_{\text {e}}$ represent the magnitude of the vector potential, the electron charge and the electron rest mass, respectively.

**Figure 1:**
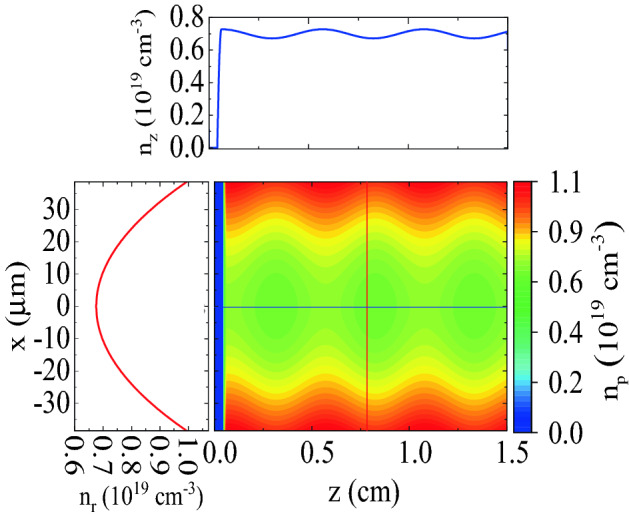
Heatmap showing the plasma density of the corrugated plasma channel. The curve at the top (blue) and the curve on the left (red) are its horizontal on-axis and vertical line-outs, respectively.

Figure 1 shows how the plasma density of the corrugated channel varies with position. A parabolic density ramp of length $L_{\text {ramp}}=200$ μm connects the vacuum to the main plasma channel. The plasma density of the channel varies sinusoidally in the longitudinal direction and parabolically in the transverse direction:

1 $$\begin{aligned} n_{\text {p}}(x,z)=n_{\text {p0}}[1+\delta \sin (k_{\text {m}}z)](1+n''_0 x^{2}/2)]. \end{aligned}$$

Here, $n_{\text {p0}}=7 \times 10^{18}$ $\text {cm}^{-3}$ is the average on-axis plasma density and $\delta =0.04$ is the amplitude of the sinusoidal modulation normalized to $n_{\text {p0}}$. The theoretical investigation of QPM-LWFA assumes that $\delta \ll 1$^41^. In simulations, an excessively small $\delta$ causes serious problems. That is because by decreasing $\delta$, the channel length as well as the initial distance between the laser pulse and the bunch must be increased if the energy gain is to remain unchanged. Therefore the simulations would require more run time and memory. The modulation period is $\lambda _{\text {m}}=5$ mm, and $k_{\text {m}}\equiv 2\pi /\lambda _{\text {m}}$. The quantity $n''_0$ determines the curvature of the plasma channel, and is related to the so-called “channel radius”, $w_{\text {ch}}$, with $n''_0=2 c^2 m_{\text {e}} / \pi e^2 n_{\text {p0}} w^4_{\text {ch}}$. The channel radius is set equal to the waist radius of the pulse, $w_0$, so that the pulse propagates optimally. It is important to note that the chosen value for $\lambda _{\text {m}}$ matches the dephasing length of the waveguide, $L_{\text{ d }}=2\lambda _{\text {p0}}^{3}\lambda _{\text {L}}^{-2}(1+8/k_{\text {p0}}^{2}w_{\text {ch}}^2)$, where $k_{\text {p0}}=\omega _{\text {p0}}/c$ with $\omega _{\text {p0}}$ being the plasma frequency at a plasma density of $n_{\text {p0}}$, and $\lambda _{\text {p0}}=2\pi /k_{\text {p0}}$. When the condition $\lambda _{\text {m}}=L_{\text {d}}$ holds, the speed of the relativistic electrons almost equals the phase velocity of the $n=-1$ spatial harmonic. As a result, this individual spatial harmonic can accelerate the electron beam over a distance many times longer than the dephasing length^41^.

**Figure 2:**
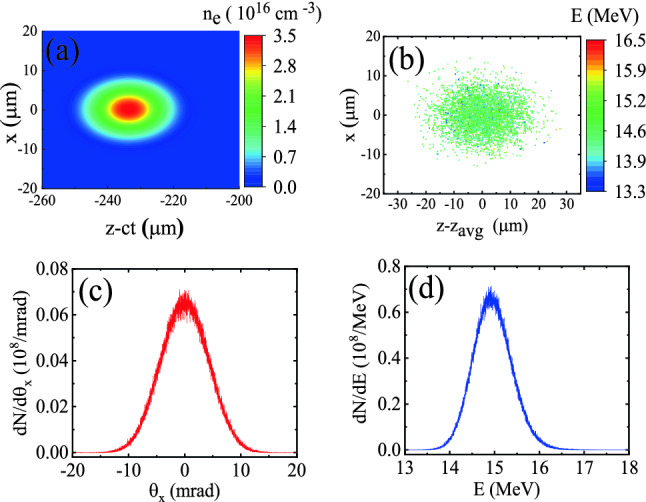
(**a**) Density distribution of a 15 MeV bi-Gaussian witness electron beam with the peak density $n_{\text {b0}}=3.5 \times 10^{16}$ $\text {cm}^{-3}$, which is initially placed 200 μm behind the peak of the laser pulse. The radii of the injected bunch in the longitudinal and transverse directions are $\sigma _{z}=8$ μm and $\sigma _{x}=4$ μm, respectively; (**b**) Spatial distribution of the electron bunch injected in the simulations; (**c**) Distribution of the inclination angle $\theta _x$; (**d**) Corresponding energy spectrum.

At the start of each simulation, the front edge of the laser pulse is located exactly where the ramp begins and a quasi-monoenergetic bi-Gaussian electron beam is placed behind the pulse in the vacuum region. The initial distance of the beam from the peak of the laser pulse is chosen to be 200 μm, so that the beam coincides with the peak of the co-moving envelope of the $n=-1$ spatial harmonic and the energy gain is maximized. The longitudinal and transverse radii of the electron beam are $\sigma _{z}=8$ μm and $\sigma _{x}=4$ μm, respectively, its charge is $q_{\text {b}}=11$ pC and it has a peak density of $n_{\text {b0}}=3.5 \times 10^{16}$ $\text {cm}^{-3}$. The electrons comprising the bunch have an average initial energy of 15 MeV. Figure 2a,b display the density distribution and the spatial distribution of the witness bunch in the z-x plane. The energy spectrum of the electron bunch is plotted in Fig. 2d, exhibiting the assigned FWHM energy spread of 7%. Figure 2c shows the inclination angle $\theta _x=\tan ^{-1}({P_x}/{P_z})$, where $P_z$ and $P_x$ represent the longitudinal and transverse particle momenta, respectively. The mean divergence angle of the bunch is only 3.4 mrad. For the bunch described above, the transverse normalized rms emittance is initially calculated to be $\varepsilon _{\text {N},x}=0.5$ $\pi$-mm-mrad according to the following definition:

2 $$\begin{aligned} \varepsilon _{\text {N},x}=\frac{1}{m_0 c}\sqrt{\text {var}(x) \text {var}(P_x)-(\text {cov}(x,P_x))^2}, \end{aligned}$$

where $\text {var}(x)$, $\text {var}(P_x)$ and $\text {cov}(x,P_x)$ respectively denote the variance of the positions, the variance of the momenta, and their covariance, in the transverse direction. When post-processing the raw outputs of the simulations to calculate distributions and quantities like energy spectrum and emittance, only those particles which are not farther from the axis than 15 μm are taken into account. This is comparable to placing a collimator at the end of the waveguide in an experiment. By increasing this radius in post-processing, emittance is increased, but our investigation show that the general conclusions of this paper are independent of the radius used in post-processing.

## Results

**Figure 3:**
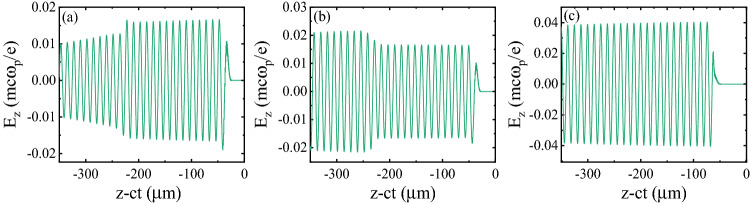
Snapshots illustrating the on-axis longitudinal wakefield, $E_z$, generated by a 30 fs laser pulse at three axial distances of (**a**) 1.4 mm, (**b**) 2.2 mm, and (**c**) 1.2 cm, corresponding to propagation times of 4.6 ps, 7 ps, and 40 ps, respectively. The wakefield is normalized to the wave-breaking field $E _{\text {WB}} = m_{\text {e}} \omega _{\text {p0}}c/e$, which is close to 254 GV/m here. The effect of beam loading on the longitudinal field can be obviously seen.

First of all, we explore the wakefield dynamics and its properties over an axial distance of 12 mm. This is shown in Fig. 3, where a sequence of line-outs of the accelerating field is displayed at three axial distances of 1.4 mm (4.6 ps), 2.2 mm (7 ps), and 1.2 cm (40 ps). We performed a detailed investigation of bunch dynamics in our previous work^42^. The electron bunch injected in the plasma waveguide is several times longer than the plasma wavelength, so it is sliced into several microbunches due to overlap with multiple accelerating and focusing areas inside the wakefield. As can be seen in Fig. 3, the amplitude of the longitudinal wakefield increases with time through the longitudinal compression of the pulse and growth of its intensity^42–44^. As the witness electron beam moves inside the plasma channel, it experiences the superposition of the laser-driven wakefield and the self-field induced by the beam itself. Therefore, the bunch directly influences the accelerating field it experiences. The self-field of the high loaded bunch strongly deforms the wakefield in a way that the accelerating gradient becomes variable along the bunch. At $t=4.6$ ps and $t=7$ ps the beam loading effect is most clearly visible locally within the moving simulation window where the injected bunch is located. At $t=4.6$ ps, the linear superposition of the self-field of the bunch and the laser-driven wakefield leads to a significant reduction in the accelerating field along the bunch. As time progresses, the bunch electrons slip with respect to the wakefield. As a result, at $t=7$ ps the wake from the accelerated bunch is in phase with, and thus boosts the wake generated by the driver laser pulse. Therefore, the electrons experience a greater accelerating field. At $t=40$ ps, a large portion of low-energy electrons are already scattered away from the axis and the self-field of the bunch is very weak. As a result, the electrons only feel the accelerating field due to the laser pulse.

In the following subsections, we investigate the influence of the pulse duration and laser envelope asymmetries on the final bunch properties in order to understand how the QPM-LWFA process can be optimized by modifying those quantities.

### Effect of the laser pulse duration

To understand the influence of the laser pulse duration on the performance of QPM-LWFA, three different durations of $\sigma _{\text {FWHM}}=20$ fs, $\sigma _{\text {FWHM}}=30$ fs and $\sigma _{\text {FWHM}}=40$ fs are assigned to 0.5 TW, 800 nm pulses in a series of simulations.

**Figure 4:**
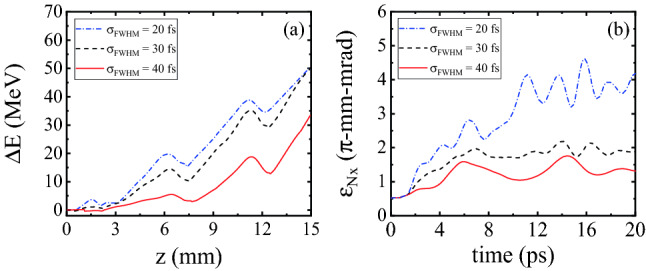
(**a**) Maximum energy gain $\Delta E$ of a witness bunch with average initial energy of $E_{0}=15$ MeV injected at optimal initial distance from the driving laser pulse, plotted versus the acceleration distance for three pulse durations at a fixed peak pulse power of 0.5 TW. The blue, black, and red colors denote the 20 fs, 30 fs, and 40 fs pulse durations, respectively; (**b**) The corresponding bunch emittance $\varepsilon _{\text {N},x}$ as a function of the propagation time.

Figure 4a shows the variations of the maximum energy gain, $\Delta E$, versus the distance, *z*, for an electron bunch initially placed at the optimum position. It can be seen that reducing the pulse duration from 40 fs to 30 fs leads to an increase in the maximum energy gain. This is due to the fact that by decreasing the pulse duration the front edge of the laser pulse interacts with the plasma electrons with an increased ponderomotive force, $F_{\text {N}} \propto \nabla E^{2}$, which generates stronger wakefields. Similarly, by decreasing the pulse duration from 30 fs to 20 fs, the generated wakefields grow further in strength. The length of a pulse with a duration of 20 fs is almost half the average on-axis plasma wavelength of the plasma channel used in the simulations. It is well known that under this condition, the amplitude of the excited wakefield is close to its maximum^2^.

Comparing the curves of maximum energy gain associated with 20 fs and 30 fs pulses in Fig. 4a, it can be observed that the maximum yielded energy is almost always higher for the 20 fs pulse, but at the end, i.e. after 1.5 cm of acceleration along the corrugated channel, it reaches $\Delta E=50.5$ MeV for both pulse durations. As we mentioned in the paragraph above, the larger ponderomotive force of the 20 fs pulse generates stronger wakefields compared to the 30 fs pulse. However, due to the stronger pump depletion of the 20 fs pulse, near the end of its propagation it excites weaker wakefields compared to the 30 fs pulse, which loses its initial energy at a lower rate. This explains why eventually the maximum energy gain is the same for the two pulse durations though it is larger for the 20 fs earlier. Since in QPM-LWFA the laser pulse is expected to propagate over a long distance, the dependence of the pump depletion length on pulse duration should be taken into account. In the linear regime of LWFA, the pump depletion length, $L_{\text {pd}}$, can be estimated by^23^

3 $$\begin{aligned} L_{\text {pd}}=\frac{c\,\sigma _{\text {FWHM}}\,\omega _{\text {L}}^2 }{\omega _{\text {p0}}^2 a_{0}^{2}}. \end{aligned}$$

To summarize, increasing the pulse duration above a certain value, which is quite close to one-half of the plasma period, decreases the efficiency. As shown in Fig. 4a, after 1.5 cm of propagation the acquired energy gain for the pulse with the initial duration of $\sigma _{\text {FWHM}}=20$ fs is about 1.5 times higher compared to the pulse with $\sigma _{\text {FWHM}}=40$ fs.

Figure 4b shows how the transverse emittance of the bunch, $\varepsilon _{\text {N},x}$, evolves over a period of 20 ps, corresponding to an axial distance of 5.2 mm or one dephasing length. Because the length of the bunch is longer than the plasma wavelength, some off-axis bunch electrons experience focusing fields and some experience defocusing fields, depending on their injection phases. The electrons located in the defocusing regions of the wakefield, are pushed away from the axis, leading to a growth of emittance. The remaining electrons, which are in the focusing regions, oscillate transversely around the axis, resulting in some oscillations in emittance. Additionally, whenever some group of electrons leave the defocusing fields and enter the focusing fields, the emittance is abruptly reduced, provided that the reduction due to the transverse focusing fields is not compensated entirely by the electrons moving into the defocusing fields. The combination of these effects causes the emittance to grow on the whole but also oscillate on a lower time scale. It can be seen in Fig. 4b that for the laser pulse with the lowest duration, the increase in the transverse emittance is highest. This is because the stronger ponderomotive force of a shorter pulse generates stronger transverse wakefields affecting the bunch electrons, thus leading to greater emittance growth. According to Fig. 4b, the emittance growth for the 20 fs laser pulse is about twice as high as for the 40 fs pulse. These results demonstrate that by using a pulse of lower duration, the transverse features of the bunch are degraded in spite of the enhancement of the maximum energy gain.

### Effect of the laser pulse shape

**Figure 5:**
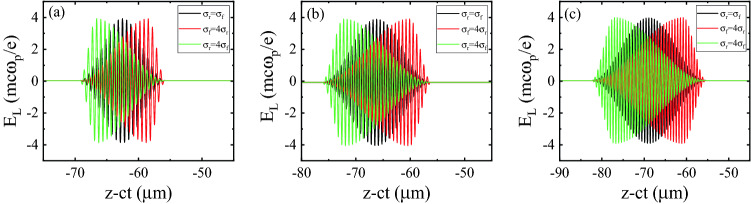
Different initial temporal profiles for the (**a**) 20 fs, (**b**) 30 fs and (**c**) 40 fs pulses, respectively. The red, black, and green colors denote the positive skew ($\sigma _{\text {f}}$ = 4 $\sigma _{\text {r}}$), the symmetric ($\sigma _{\text {r}}$ = $\sigma _{\text {f}}$ = $\sigma _{\text {0}}/2$), and the negative skew ($\sigma _{\text {r}}$ = 4 $\sigma _{\text {f}}$) cases, respectively.

Our aim here is to study how the laser pulse shape (specifically, its skewness) affects the quality of the externally injected electron beam traveling through the corrugated plasma channel. For each driving laser pulse length, we consider three different initial temporal profiles: a symmetric sine-squared pulse and two asymmetric pulses, one with a sharp rising front and a slow fall (positive skew) and the other with a gentle rising front and a sharp fall (negative skew). The initial asymmetry introduced in the pulse shape is clearly visible in Fig. 5a–c, in which $\sigma _{\text {f}}$ and $\sigma _{\text {r}}$ denote the fall edge and rise edge of the laser pulse, respectively, and its full length equals $\sigma _{\text {0}}=\sigma _{\text {r}}+\sigma _{\text {f}}$.

**Figure 6:**
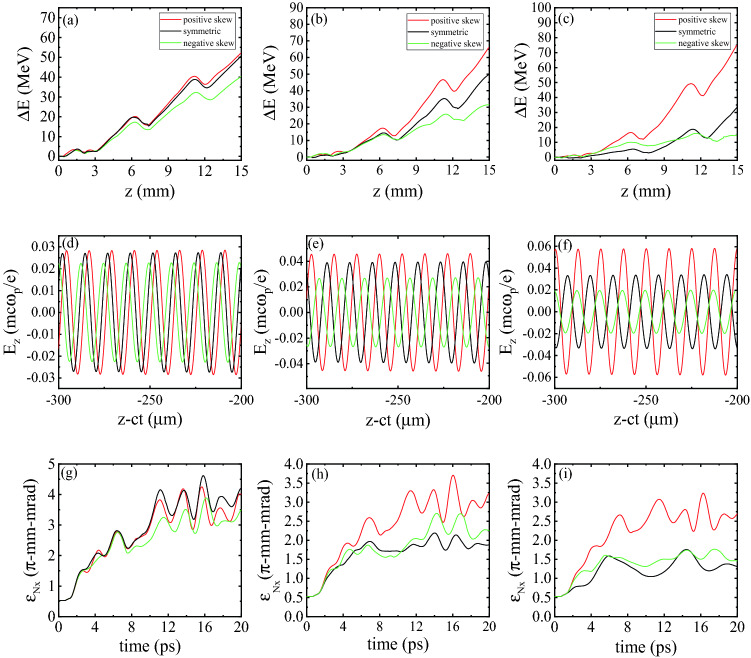
(**a**–**c**) show the variation of the maximum energy gain $\Delta E$ as a function of axial distance for (**a**) 20 fs, (**b**) 30 fs and (**c**) 40 fs pulses; (**d**–**f**) show the corresponding longitudinal wakefields at $t=40$ ps; (**g**–**i**) show the corresponding transverse emittances, $\varepsilon _{\text {N},x}$, as a function of the propagation time. The red, black, and green colors denote the positive skew, the symmetric, and the negative skew cases, respectively. In the simulations, the laser pulse is linearly polarized with a peak amplitude of $a_{0} = 0.25$, a wavelength of 800 nm, and a focal spot size (FWHM) of $w_{0} = 15$ μm.

Figure 6a–c shows the changes in the maximum energy gain of the bunch electrons as the bunch travels in the plasma channel. It is clear that the maximum energy gain can be improved using asymmetric laser pulses with a fast-rising leading edge for all of the three pulse durations. The line-outs of the normalized longitudinal wakefield at $t=40$ ps, corresponding to an axial distance of 1.2 cm, for all the three profiles are shown in Fig. 6d–f. It is seen that the amplitude of the wakefield increases with the gradient of the rising part. So, by using a pulse that has positive skew, higher energy gain can be achieved. The stronger wakefield excited by the positive-skew pulses is a result of the larger ponderomotive force that their leading edges exert. When $\sigma _{\text {FWHM}}=20$ fs, which is almost half the plasma period, the accelerating field and maximum energy gain in all three profiles show little difference, as can be seen in Fig. 6a,d. It is clear that the positive-skew and symmetric pulses perform comparably, while the energy gain is perceptibly less for the negative-skew pulse due to the weak ponderomotive force available from a pulse with a slow-rising front. For a longer pulse duration of 30 fs, the differences in the accelerating fields and maximum energy gains for the three pulse profiles are bigger. As can be seen in Fig. 6e, the positive-skew laser pulse generates the wakefield with the highest amplitude, compared to the other two temporal profiles. According to Fig. 6b, the differences in the maximum energy gain up to a distance of 5 mm are not considerable, but further propagation of the laser pulse increases the difference. These differences arise because of the longitudinal compression of the pulses, which is greater for the positive skew pulse. As a consequence of pulse compression, the peak amplitude of the pulses increases, resulting in enhanced wakefield amplitude and improved energy gain. After 1.5 cm of acceleration inside the plasma channel, the maximum energy gain for the asymmetric laser pulse with a fast rise time is about 30% higher than for the temporally symmetric pulse. The maximum energy gain and accelerating field for the pulse duration of $\sigma _{\text {FWHM}}=40$ fs are illustrated in Fig. 6c,f. At the end of the simulations, the maximum energy gain for the positive skew pulse is more than two times larger than for the symmetric pulse. A comparison with Fig. 6a,b shows that the 40 fs pulse with the sharp steepening of its leading front is favorable for attaining higher electron bunch energies, with the energy gain reaching 75 MeV.

Figure 6g–i displays the time evolution of the transverse emittance for the positive skew, negative skew, and symmetric pulses. As shown in these figures, the asymmetric pulse with positive skew causes greater transverse bunch emittance due to the stronger focusing and defocusing wakefields it generates. Therefore, using an asymmetric laser pulse with a fast rise time improves the maximum energy gain of the electron beam, but it also increases the final transverse emittance of the bunch, $\varepsilon _{\text {N},x}$. One can see that initially, the transverse emittance is almost the same for the three profiles, as shown in Fig. 6g–i. However, the evolution of the driving laser pulse during its propagation in the plasma has an important role and leads to significant emittance growth for the positively skewed pulse. Additionally, increasing the laser pulse duration results in larger differences in the transverse emittance among the cases associated with the three profiles.

**Figure 7:**
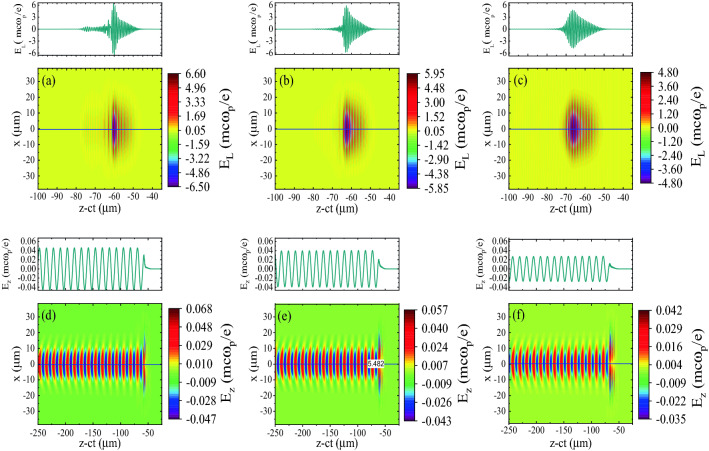
(**a**–**c**) show the transverse field of the laser pulses, $E_L$, along with the horizontal on-axis line-outs for (**a**) the pulse with positive skew, (**b**) the symmetric pulse, and (**c**) the pulse with negative skew, at the time $t=40$ ps; (**d**–**f**) show the corresponding wakefields along with the horizontal on-axis line-outs. Each pulse initially has a peak amplitude of $a_{0} = 0.25$, with a duration of 30 fs, and a spot size of $w_{0} = 15$ μm.

As discussed above, at the early phase of laser-plasma interaction, the sharp rising front of the positively skewed pulse generates a larger wakefield compared to the symmetric pulse and the negatively skewed pulse. As time passes, the nonlinear evolution of the laser pulses propagating in the channel plays an essential role in the wakefield evolution. Figure 7a–c displays the transverse field, $E_\text {L}$, of three 30 fs laser pulses with different initial temporal profiles at $t=40$ ps. It can clearly be seen that each pulse undergoes longitudinal compression and its amplitude increases as it propagates through the plasma. This can be explained by using photon kinetic theory. When a laser pulse propagates through a plasma, its ponderomotive force causes a variation in the electron density along with the pulse. This variation in the electron density together with the dependence of the relativistic mass of the plasma electrons on the local intensity of the pulse leads to a nonlinear gradient in the refractive index along with the pulse. In our simulation with the symmetric pulse, the refractive index increases monotonically from the front of the pulse backwards throughout most of the pulse. In response to this laser-induced gradient in the refractive index, most of the photons are downshifted by various degrees. Since the group velocity declines with decrease in frequency ($v_{\text {g}}\approx c[1-\omega ^2_{\text {p}}/2\omega ^2]$), the redshifted photons are decelerated. The largest deceleration initially occurs near the center of the pulse as the photons there are redshifted most. So the pulse stretches in the front and compresses in the back. The positively skewed and negatively skewed pulses evolve similarly. Nevertheless, there are also important differences, because the initial pulse profile and the exact profile of the variations in the local refractive index differ in each case. Figure 7a–c shows that pulse compression and amplification are most intense for the positively skewed pulse and weakest for the negatively skewed pulse. Therefore, as can be seen in Fig. 7d–f, the positively skewed pulse generates the strongest wakefield.

## Discussion

Here, we study how QPM-LWFA can be modified by controlling the duration and waveform of the driving laser pulse. The acceleration of externally injected electron bunches traveling in the plasma wakefield driven by asymmetric laser pulses in an axially-modulated plasma channel is inspected by performing 2D PIC simulations. To investigate the influence of the laser pulse duration, we consider a symmetric driving laser pulse profile with three different pulse durations of $\sigma _{{FWHM}}=20$ fs, $\sigma _{\text {FWHM}}=30$ fs, and $\sigma _{\text {FWHM}}=40$ fs under a fixed peak pulse power of $P_{\text {L}}=0.5$ TW. The results show that applying a duration of $\sigma _{\text {FWHM}}=20$ fs which is around half the plasma period, results in the highest maximum energy gain of the beam. By increasing the pulse duration to 30 fs, the ponderomotive force at the front edge of the pulse decreases, which leads to a reduction of the wakefield amplitude and also energy gain. At the end of the plasma channel, the difference in energy gain for these two pulse durations approaches zero, due to the faster depletion of the 20 fs laser pulse. On the other hand, the stronger focusing and defocusing fields generated by the shorter pulse result in an increase in the transverse emittance. Furthermore, simulations are conducted to study the effect of the skewness of the pulse shape on the final properties of the accelerated bunch. In comparison to temporally symmetric laser pulses and asymmetric pulses with a slow rise time, asymmetric laser pulses having fast rise time can generate larger wakefields, regardless of the pulse duration. As a result, the maximum energy of the electron beam can be enhanced using asymmetric laser pulses with a fast rise time. For the laser pulse shorter than the plasma wavelength with a duration of 20 fs, the effect of the skewness of the pulse shape is weak and the generated wakefields corresponding to different skewness modes do not demonstrate much difference. For longer pulse lengths, however, the differences become more significant. As an example, at a duration of 40 fs, the final maximum energy gain for the positive skew is almost twice larger than for the symmetric pulse profile. The simulation results show that the positive skew pulse with the duration of 40 fs yields the best maximum energy gain, which is $\Delta E=75$ MeV after 1.5 cm of acceleration for a peak pulse power of 0.5 TW. Finally, the results show that early in the simulation, when SPM and other nonlinear effects have not altered the pulse significantly yet, the wakefield amplitude for the positively skewed pulse is larger than for the other two cases due to the larger ponderomotive force available from the fast-rising front pulse. As time passes, nonlinear effects cause the pulses to stretch in the front and compress in the back, thus increasing their maximum intensity. The largest increase in the maximum intensity occurs in the positive skew pulse, so that it keeps generating a stronger wakefield compared to the other two pulses. The result is that the maximum energy gain is highest for the positive skew pulse. In addition, it is found that the increase in the transverse emittance of the bunch during each simulation is highest for positive skew laser pulses. The difference in the growth of transverse emittance between the positive skew pulse and the two other cases becomes more significant at larger pulse durations. Thereby, although positive skew pulses are favorable for achieving higher electron beam energies, they degrade the transverse characteristics of the bunch due to the stronger transverse wakefields generated by them.

## Data Availability

The data that supports the results of this study is available from the corresponding author upon reasonable request.
